# Extraction of DNA from micro-tissue for bat species identification

**DOI:** 10.1080/23802359.2018.1484261

**Published:** 2018-07-10

**Authors:** Thaís Fernandes Mendonça Mota, Thomaz Mansini Carrenho Fabrin, Luciano Seraphim Gasques, Henrique Ortêncio Filho, Alberto José Prioli, Sônia Maria Alves Pinto Prioli

**Affiliations:** a Programa de Pós-Graduação em Biologia Comparada, Universidade Estadual de Maringá, Maringá, Brazil;; b Programa de Pós-Graduação em Ecologia de Ambientes Aquáticos Continentais, Universidade Estadual de Maringá, Maringá, Brazil;; c Departamento de Ciências Biológicas, Universidade Paranaense – UNIPAR, Umuarama, Brazil;; d Departamento de Ciências, Universidade Estadual de Maringá, Maringá, Brazil;; e Nupélia, Universidade Estadual de Maringá, Maringá, Brazil;; f Departamento de Biotecnologia, Genética e Biologia Celular/Nupélia, Universidade Estadual de Maringá, Maringá, Brazil

**Keywords:** *COI*, DNA barcode, uropatagium, Chiroptera, *Histone H3*, mitochondrial

## Abstract

Bat populations are declining worldwide. Accurate identification is essential to promote species’ conservation. However, minimal morphological differences and a high rate of cryptic species make identification difficult, unless voucher specimens are kept, a controversial issue today. The objective of this work was to standardize a method of extracting non-lethal DNA using bats’ uropatagium micro-tissue, aiming the molecular identification of species that occur in the region of Maringá PR. The method standardized was efficient, and does not cause serious damage to bats. For future field studies, collection of micro-tissue and morphometry of the specimens will be sufficient for accurate identification.

## Introduction

Chiroptera presents over 1300 recognized species (Tsang et al. 2016) and although they perform important ecological functions (Boyles et al. 2011), at least 16% of the species are threatened (Voigt and Kingston 2016, IUCN 2017). Among the measures in the reversion of this is a correct identification of the species, mainly performed through morphological analysis (Francis et al. 2010), or either by acoustic analysis of echolocation, which presents practical problems (Rydell et al. 2017), or by molecular techniques (Clare 2011; Pavan and Marroig 2016).

Most species present minimal morphological differences, overlapping measurements (Miranda et al. 2011) and a high rate of cryptic species (Clare et al. 2007), highlighted by molecular studies (Dool et al. 2016; Gager et al. 2016). Thus, field identifications are questionable unless voucher specimens are kept, which is oftentimes hampered by environmental licences or ethical concerns (Wilson et al. 2014). It is still a controversial issue in modern biology (Russo et al. 2017) and raises concerns about unnecessary collections of organisms (Corthals et al. 2015), having as an aggravating that vouchers could in many cases be safely replaced with images and molecular studies (Corthals et al. 2015; Raupach et al. 2016). The DNA barcode has been widely used as a fast and accurate tool in the identification and differentiation of the species (Clare et al. 2011; Wilson et al. 2014).

In some studies, non-lethal methods for molecular research have been used, such as fecal samples, buccal swab, blood (Walker et al. 2016) and the wing and tail membrane (Faure et al. 2009; Wilson et al. 2014). Uropatagium tissue has been recommended for collection because it heals quickly and it provides a high quantity of DNA (Faure et al. 2009). Many field studies pierce these membranes to mark the animal by discarding the surplus micro-tissue. The objective of this work was to standardize a method of extracting non-lethal DNA from a bats’ uropatagium micro-tissue, aiming the molecular identification of species that occur in the region of Maringá PR.

## Methodology

### Collection of material

The collections were carried out by the Grupo de Estudos em Ecologia de Mamíferos e Educação Ambiental (GEEMEA) in fragments of Atlantic Forest in the city of Maringá, Paraná, Brazil (23°25′58″S 51°58′06″W). Collections were performed using mist. Individuals were measured and identified according to the criteria of Gregorin and Taddei (2002). The tissue (1 mm^2^) was extracted from the uropatagium using a disposable biopsy punch (Disposable Biopsy Punch, 1 mm – Miltex^®^), afterwards it was stored in a microtube with absolute ethanol. The tubes were kept at −20 °C until DNA extraction.

Uropatagium tissues were collected from 50 specimens, which were released and observed flying without difficulties. The bats captured were attributed to 13 species (Table 1). Specimens were collected under a scientific collecting permit (Sisbio 55121-2, process 3097240916).

**Table 1.** Number of samples collected by Chiroptera species in the northwestern region of Paraná.

Species	Number of samples
*Artibeus fimbriatus* (Gray, 1838)	5
*Artibeus lituratus* (Olfers, 1818)	7
*Artibeus obscurus* (Schinz, 1821)	3
*Artibeus planirostris* (Spix, 1823)	4
*Carollia perspicillata* (Linnaeus, 1758)	6
*Molossops neglectus* (Williams and Genoways, 1980)	1
*Molossus molossus* (Pallas, 1766)	7
*Molossus rufus* (E. Geoffroy, 1805)	6
*Myotis nigricans* (Schinz, 1821)	1
*Phyllostomus hastatus* (Pallas, 1767)	2
*Platyrrhinus lineatus* (E. Geoffroy, 1810)	2
*Sturnira lilium* (E. Geoffroy, 1810)	5
*Vampyressa pusilla* (Wagner, 1843)	1

### Extraction of DNA

The DNA was extracted with the ReliaPrep ™ column extraction kit (ReliaPrep ™ gDNA Tissue Miniprep System – Promega), with three different protocols: (I) 10 extractions of DNA from uropatagium were performed according to the manufacturer’s instructions; (II) 10 extractions of DNA from uropatagium were performed. Initially the tissue was rehydrated in TE (TRIS HCl pH 8.0 10 mM/EDTA pH 8.0 1 M) for 1 h, then the steps recommended by the manufacturer were performed, but the DNA was re-suspended in only 50 μL of Nuclease-Free Water; and (III) 30 individuals were used in extraction III, following the same steps as extraction II, but the tissue sample was triturated with slide prior to incubation at 56 °C for 3 h.

### PCR and DNA sequencing

Partial sequences of the mitochondrial cytochrome C oxidase I gene (*COI*) were obtained with the primers described by Ivanova et al. (2007). For confirming the success of the extraction, samples that did not amplify with *COI* were subjected to amplification using primers from part of the *Histone H3* nuclear gene (Colgan et al. 1998). Finally, samples that did not amplify with the *Histone H3* were discarded.

The PCR reactions (25 μL) were carried out containing Tris-KCl (20 mM of Tris-HCl, pH 8.4 and 50 mM of KCl), 1.5 mM of MgCl2, 2.5 mM of each primer, 0.1 mM of each dNTP, 1 μL Taq of DNA polymerase, mold DNA and water. Since it was not possible to quantify the DNA extraction by virtue of their small amount, two different volumes of template DNA were tested: the first PCR (1) with 2 μL and the second (2) with 5 μL.

The temperatures used in the PCR followed an initial cycle at 94 °C, 1 min; five cycles at 94 °C, 30 s, 50 °C, 40 s, and 72 °C, 1 min; followed by 35 cycles at 94 °C, 30 s, 55 °C (*COI*) – 58 °C (*Histone H3*), 40 s and 72 °C, 1 min, followed by a final extension for 10 min at 72 °C. Amplicons of the *COI* were purified according to Rosenthal et al. (1993), and these were subjected to BigDye Terminator Cycle (Foster City, CA) sequencing and DNA sequencing was performed using a Applied Biosystems 3730XL(Carlsbad, CA), both according to manufacturer’s instructions. 

### Data analysis

The genetic distance matrix, sequences alignment and model selection were performed in the MEGA 7 program (Kumar et al. 2016). A phylogenetic network was constructed using the NeighborNet method (Bryant and Moulton 2004), with SplitsTree4 (Huson and Bryant 2006). The phylogenetic trees were constructed using raxmlGUI (Silvestro and Michalak 2012) and MrBayes 3.2 (Ronquist et al. 2012), considering the statistical methods of maximum likelihood and Bayesian inference, respectively. The BLAST method was used to species identification using *COI* (Ross et al. 2008).

## Results

The samples extracted in the I and II protocols showed no amplification. However, the III protocol (30 samples) resulted in 23 samples amplified with *COI*, and 70% were obtained from PCR reaction with 2 μL of template DNA. Seven samples that did not amplify with the *COI* were tested with *Histone H3*; of these, three amplified, confirming the success of the extraction.

Partial sequences of *COI* with 595 bp were obtained from 23 specimens, distributed in 13 species, with 21 different haplotypes. Specimens attributed to different *Artibeus* species shared haplotypes (specimens 224 and 217; specimens 382 and 207).

The interspecific variation in *Artibeus* was 0.72%. Some species collected showed similarity of 100% with species deposited in GenBank (Table 2).

**Table 2.** Identification of morphologic and identification of the species using similarity analysis with the BLAST algorithm, *COI* sequences available in GenBank and of *COI* sequences obtained from DNA extracted from the bats’ uropatagium micro-tissue (*e*-value <0.0).

Specimen	Species identified in field	Morphometry (forearm in mm)	GenBank access number	BLAST – similarity of species using sequences of *COI*
280	*Artibeus fimbriatus*	37.10	MG182643	100% *Artibeus lituratus*
382	*Artibeus fimbriatus*	68.73	MG182644	100% *Artibeus lituratus*
217	*Artibeus lituratus*	69.40	MG182645	100% *Artibeus lituratus*
225	*Artibeus lituratus*	70.66	MG182646	100% *Artibeus lituratus*
224	*Artibeus obscurus*	74.24	MG182647	100% *Artibeus lituratus*
422	*Artibeus obscurus*	80.90	MG182648	100% *Artibeus lituratus*
205	*Artibeus planirostris*	72.57	MG182649	100% *Artibeus lituratus*
207	*Artibeus planirostris*	62,49	MG182650	100% *Artibeus lituratus*
218	*Carollia perspicillata*	76.75	MG182651	100% *Carollia perspicillata*
219	*Carollia perspicillata*	69.70	MG182652	100 % *Carollia perspicillata*
494	*Molossops neglectus*	38.52	MG182653	94% *Molossops temminckii* (Burmeister, 1854)
				93% *Molossops neglectus*
234	*Molossus molossus*	37.38	MG182654	98% *Molossus molossus*
235	*Molossus molossus*	37.40	MG182655	99% *Molossus molossus*
236	*Molossus rufus*	38.33	MG182656	99% *Molossus rufus*
241	*Molossus rufus*	37.02	MG182657	99% *Molossus rufus*
379	*Myotis nigricans*	71.03	MG182658	95% *Myotis nigricans*
175	*Phyllostomus hastatus*	69.73	MG182659	95% *Phyllostomus hastatus*
393	*Phyllostomus hastatus*	42.05	MG182660	95% *Phyllostomus hastatus*
197	*Platyrrhinus lineatus*	46.85	MG182661	99% *Platyrrhinus lineatus*
198	*Platyrrhinus lineatus*	44.07	MG182662	100% *Platyrrhinus lineatus*
72	*Sturnira lilium*	43.88	MG182663	100% *Sturnira lilium*
73	*Sturnira lilium*	42.33	MG182664	99% *Sturnira lilium*
414	*Vampyressa pusilla*	71.23	MG182665	100% *Vampyressa pusilla*

**Figure 1.**
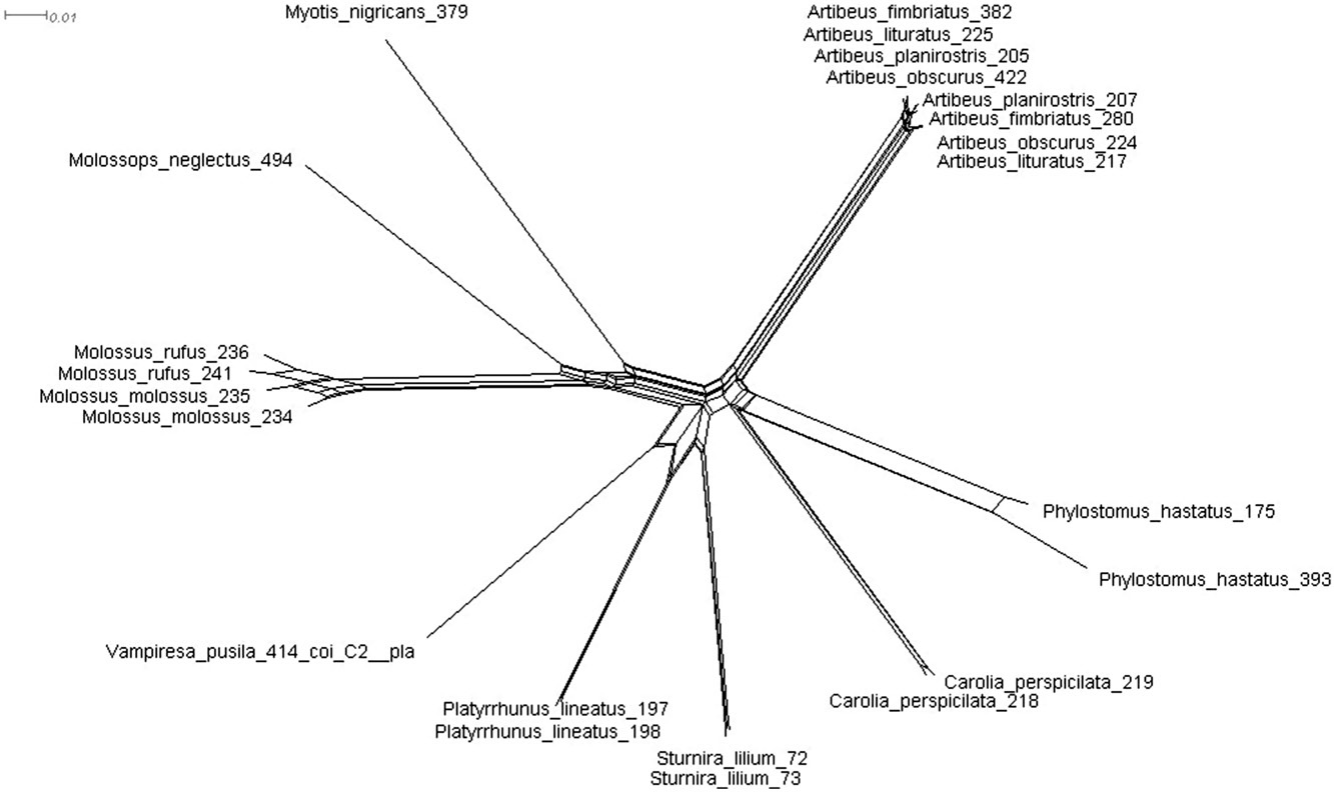
NeighborNet network of the *COI* gene from specimens of Chiroptera assigned to different species.

**Figure 2.**
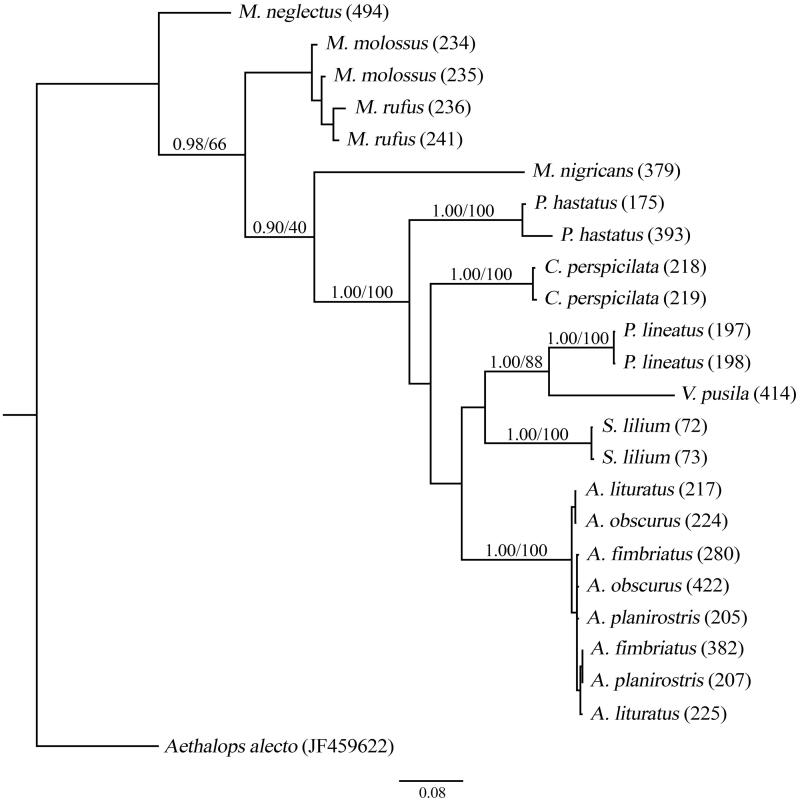
Phylogenetic tree constructed using partial fragments of the *COI* (595 bp) according to the substitution model GTR + I. Numbers above the branches represent *a posteriori* probability and bootstrap, respectively.

The NeighborNet phylogenetic network and the phylogenetic tree showed that individuals of the same species were grouped, with the exception of individuals of *Artibeus* species (Figures 1 and 2).

## Discussion

The results of this study showed that it is possible to successfully carry out DNA extraction from uropatagium micro-tissue modifying the manufacturer’s protocol, reducing the risks for the animal. Many studies use wing membrane tissue for DNA extraction; however, multiple biopsies are needed from the same animal, aiming to increase the tissue mass for extraction (Vonhof et al. 2008). This procedure causes pain and increases the bleeding potential.

The III protocol was the only one that allowed to amplify the two genes tested. Rehydration, triturated of the sampled material and increase in incubation time may have facilitated the extraction process, since studies have already demonstrated that in membrane extractions of flight it is common that the tissue is not completely digested (Faure et al. 2009), besides most of the samples were amplified with less amount of template DNA.

The initial identification of specimen 494 was that of *Molossops neglectus*. However, according to the *COI* gene, the specimen presented greater genetic similarity to *M. temminckii*, a species that is also registered in Paraná (Miretzki and Margarido 1999). *M. neglectus* and *M. temminckii* are supported as sister species (Peters et al. 2002), justifying the high genetic similarity evidenced by the *COI* gene. This fact reinforces the thesis of errors in the identification of specimens at the capture site.

The specimens attributed to *Artibeus fimbriatus*, *A. obscurus* and *A. planirostris* presented 100% similarity to *A. lituratus*, which was expected since specimens attributed to different *Artibeus* species shared haplotypes. Several studies with bats indicate that the average intraspecific variation does not exceed 2% (Bradley and Baker 2001; Francis et al. 2010; Clare 2011; Clare et al. 2011).

*Artibeus* presents difficulties in species identification based on morphological characters. There are overlapping measurements for *A. fimbriatus*, *A. obscurus* and *A. planirostris* and *A. lituratus*, and because they are morphologically very similar, they are commonly confused (Araújo and Langguth 2010). Thus, both the specimens used in this work and the sequences of the specimens that were deposited in GenBank may have been misidentified. Of the seven specimens that were not conclusively identified, six were *Artibeus*.

Excluding the *Artibeus* specimens, 93.3% of the sequenced samples were conclusively identified. In studies using barcode DNA in bats, 96.6% of the individuals sampled were correctly identified (Borisenko et al. 2008). Therefore, we conclude that DNA barcode is efficient in identifying bat species in the neotropical region.

It is important to emphasize that for the extraction of DNA, it was not necessary to euthanize the animal, and it was possible to amplify a nuclear marker, indicating that other markers can be used to solve complex questions. In future field studies, only the collection of micro-tissue, photographic images and morphometry of the specimens will be sufficient for correct identification, contributing to the reduction of ethical problems in research and animal welfare.
